# The Neuronal Synuclein Disease Integrated Staging System ‐– Opportunities to advance Lewy body dementia research

**DOI:** 10.1002/alz.091416

**Published:** 2025-01-09

**Authors:** Kathleen L. Poston

**Affiliations:** ^1^ Stanford University School of Medicine, Stanford, CA USA

## Abstract

Recent advances in biomarkers, enabling the in vivo detection of pathological aggregates of alpha‐synuclein (asyn), allow a shift from a clinical to a biological definition of Parkinson’s disease (PD) and dementia with Lewy bodies (DLB). The newly proposed “Neuronal alpha‐Synuclein Disease (NSD)” is defined by the presence of pathologic neuronal (n‐asyn) species detected in vivo (S), irrespective of any specific clinical syndrome. Additional biological anchors include dopaminergic neuronal dysfunction (D). This biological definition forms the basis for the NSD Integrated Staging System (NSD‐ISS), rooted in the biological anchors (S and D) and the degree of functional impairment caused by clinical signs/symptoms.

Stage 1 and 2 are marked by S alone (Stage 1A or 2A), or both S and D (Stage 1B or 2B), differentiated by absence of signs/symptoms (stage 1) or appearance of subtle signs/symptoms without functional impairment (stage 2). Critically, these patients could progress to Stage 3 by developing a functional impairment due to cognitive or motor or other non‐motor impairments. Stages 2B‐6 require both S and D, with Stages 3‐6 defined by their stage‐specific increases in functional impairment.

The NSD definition and NSD‐ISS framework will enhance drug development and clinical trial efficacy by facilitating the selection of a homogeneous target population with biological consistency, which is particularly relevant to drug development in Lewy body dementias. Also critical to research in Lewy body dementias, NSD could inform how and when to co‐enroll patients with dementia regardless whether they initially presented with motor or cognitive impairments. The NSD‐ISS framework will also context for considering research in Lewy body dementia patients with and without ATN biomarkers. Currently NSD‐ ISS is a research tool and is not intended for clinical use.

